# The roles of microRNAs in regulation of mammalian spermatogenesis

**DOI:** 10.1186/s40104-017-0166-4

**Published:** 2017-05-01

**Authors:** Xiaoxu Chen, Xueliang Li, Jiayin Guo, Pengfei Zhang, Wenxian Zeng

**Affiliations:** College of Animal Science and Technology, Northwest A&F University, Yangling, Shaanxi 712100 China

**Keywords:** microRNAs, RNA methylation, Spermatogenesis

## Abstract

Mammalian spermatogenesis contains three continuous and organized processes, by which spermatogonia undergo mitosis and differentiate to spermatocytes, follow on meiosis to form haploid spermatids and ultimately transform into spermatozoa. These processes require an accurately, spatially and temporally regulated gene expression patterns. The microRNAs are a novel class of post-transcriptional regulators. Cumulating evidences have demonstrated that microRNAs are expressed in a cell-specific or stage-specific manner during spermatogenesis. In this review, we focus on the roles of microRNAs in spermatogenesis. We highlight that N6-methyladenosine (m6A) is involved in the biogenesis of microRNAs and miRNA regulates the m6A modification on mRNA, and that specific miRNAs have been exploited as potential biomarkers for the male factor infertility, which will provide insightful understanding of microRNA roles in spermatogenesis.

## Background

Male fertility is dependent upon the successful perpetuation of spermatogenesis that is a highly organized process of germ cell differentiation occurring within the seminiferous tubules in the testes. Spermatogonial stem cells (SSCs) are a subset of undifferentiated spermatogonia that are capable of self-renewal to maintain the pool of SSCs or differentiation to give rise to spermatogenic lineage, thus supporting the continuous production of spermatozoa. Spermatogenesis initiates once SSCs enter differentiation process [1]. The spermatogonia go into the meiotic phase and become spermatocytes. After a long-lasting meiosis I, preleptotene spermatocytes transform into second spermatocytes and enter meiosis II to produce haploid round spermatids [2], which undergo spermiogenesis including acrosomal biogenesis, flagellum development, chromatin condensation, cytoplasmic reorganization and exclusion [3]. Ultimately, the round spermatids transform into spermatozoa, which are released into the lumen of seminiferous tubules [4].

This highly organized spermatogenesis requires accurate, spatial and temporal regulation of gene expression governed by transcriptional, post-transcriptional and epigenetic processes [5, 6]. More than a thousand of protein coding genes that are involved in the spermatogenesis have been identified [7, 8]. However, the mechanisms that mediate the expression of these spermatogenesis-related genes have not been fully uncovered. The microRNAs (miRNAs, miR), small (~22 nucleotides) single-strand noncoding RNAs, are linked to cell proliferation, differentiation and apoptosis [9–11]. Transcriptome data indicate that miRNAs are extensively transcribed during spermatogenesis. The miRNAs are differentially expressed in a cell-specific and step-specific manner ([12, 13], Chen et al. unpublished data). Some miRNAs are specifically expressed in certain type of male germ cells, while the others are universally expressed among different types of cells in the testes. Growing evidences have showed that the miRNAs are essential for male germ cell development and differentiation [14–17]. A few recent reviews have reported the roles of miRNAs in spermatogenesis and fertility [5, 6, 10, 11]. In this article, we briefly summarize the most recent progress of miRNAs in the regulation of spermatogenesis.

### miRNA biogenesis

At present, there are 1881 miRNA loci having been annotated in the human genome in the miRNA database (http://www.mirbase.org). Analysis has revealed that 1% of the human genome is miRNA genes [18–20], of which about half of miRNA genes located in the introns (intronic miRNAs) of host genes [21]. However, some intronic miRNAs exhibit low correlated expression level with their host genes. It is likely these miRNAs are transcribed from unique transcription units independent of host genes [22–24].

**Fig. 1:**
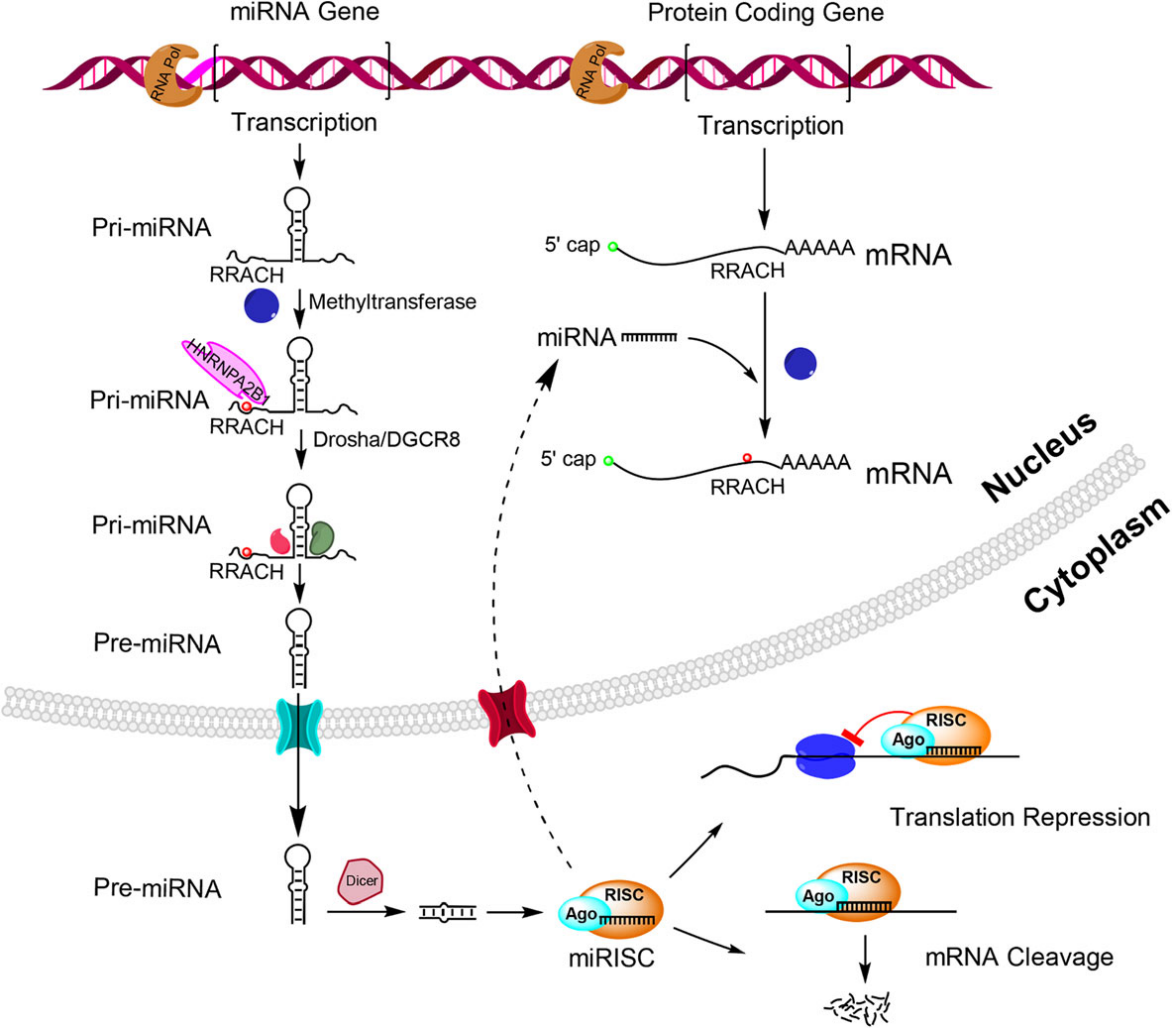
Biogenesis and function of miRNAs

The biogenesis of miRNAs is modulated at a few levels, including miRNA transcription, processing by Drosha and Dicer, RNA methylation, uridylation and adenylation (Fig. 1) [25–27]. The initial transcripts are termed the primary miRNAs (pri-miRNAs) that are variable in length from several hundreds to thousands of nucleotides [25]. The pri-miRNAs are methylated by the methyltransferase like 3 (METTL3), marking them for recognition and processing by the DiGeorge syndrome critical region 8 (DGCR8) [28]. The pri-miRNAs are thus processed by drosha ribonuclease III (Drosha) and its cofactor DGCR8 into ~ 70 nucleotides (nt) long miRNA precursor (pre-miRNAs) [29, 30]. The pre-miRNAs are then transported into the cytoplasm by exportin 5 (EXP5) in accompanied with Ran-GTP [31, 32] and cleaved by Dicer into ~22 base pair (bp) double-strands RNAs (dsRNAs) [33–35]. These dsRNAs are loaded onto an Argonaute protein (AGO) so as to form miRNA-induced silencing complex (miRISC), in which one strand of the ~22-nt RNA duplex remains in AGO as a mature miRNA, whereas the other strand is degraded [36]. Interestingly, Alarcon et al. recently reported that RNA-binding protein heterogeneous nuclear ribonucleoprotein A2/B1 (HNRNPA2B1) binds m6A-bearing pri-miRNAs, interacts with DGCR8 and thus facilitates the processing of pri-miRNAs [37]. In consistent with this, loss of HNRNPA2B1 or depletion of METTL3 led to concomitant accumulation of unprocessed pri-miRNAs and decrease of the global mature miRNAs [28, 37]. Therefore, the methylation mark acts as a key post-transcriptional modification that enhances the initiation of miRNA biogenesis.

### Mechanisms of miRNA action

Usually, a specific base-pairing between miRNAs and mRNAs induces mRNA degradation or translational repression [38]. In mammals, the overall complementarity between a miRNA and its target is usually imperfect, which allows each miRNA to potentially regulate multiple RNAs [39]. It is estimated that one miRNA may target as many as 400 genes on average [19]. Conversely, the expression of a single gene can also be modulated by multiple miRNAs [40].

Interestingly, it has been reported recently that miRNAs regulate the m6A modification in mRNAs via a sequence pairing mechanism. As a result, manipulation of miRNA expression leads to change of m6A modification through modulating the binding of METTL3 to mRNAs (Fig. 1) [41]. The m6A modification, in turn, modulates mRNA metabolism and thus is another key post-transcriptional control of gene expression [37, 42, 43]. Evidences have indicated that m6A methylation determines stem cell fate by regulating pluripotent transition toward differentiation [41, 44, 45]. Intriguingly, deficiency of ALKBH5, a m6A demethylase, leads to aberrant spermatogenesis and apoptosis in mouse testis through the demethylation of m6A on mRNAs [46].

### Functions of miRNAs in spermatogenesis

#### Conditional Dicer knockout mouse models

The overall importance of miRNA signaling for regulation of spermatogenesis has been demonstrated using conditional knockout of *Dicer* in germ cells. *Dicer1* ablation in prospermatogonia just before birth using *Ddx4* promoter-driven Cre expression led to an alteration in meiotic progression, significant increase of apoptosis in pachytene spermatocytes, a reduced number of round spermatids and morphological defects in spermatozoa [47]. Moreover, *Ngn3* is expressed endogenously in type A spermatogonia starting from postnatal d 5 [48, 49]. In the mouse model of selective deletion of *Dicer1* in type A spermatogonia by *Ngn3* promoter-driven Cre, the first clear defects were displayed in haploid round spermatids. The spermiogenesis was severely compromised [50]. Similarly, conditional depletion of *Dicer1* using the *Stra8Cre* transgene in early spermatogonia resulted in the comparable phenotype to the *Ngn3Cre*-driven *Dicer1* deletion [51, 52]. In addition, deletion of *Dicer1* in haploid spermatids using the *protamine 1 (Prm1)-Cre* transgene led to abnormal morphology in the elongated spermatids and spermatozoa [53]. But, the *Prm1Cre-Dicer1* knockout caused a less severe phenotype compared to those in which Dicer1 was deleted from prospermatogonia and spermatogonia [53].

Collectively, the earlier the ablation of Dicer occurs, the more severe side effects on spermatogenesis are found. Therefore, miRNA-mediated post-transcriptional control is an important regulator for spermatogenesis.

#### The roles of miRNAs in SSC self-renewal and differentiation

SSCs are the foundation of spermatogenesis that involves a delicate balance between self-renewal and differentiation of SSCs to ensure the lifelong production of spermatozoa. In the testes, the SSCs reside in a unique microenvironment or ‘niche’. The niche factor glial cell line-derived neurotropic factor (GDNF) is the first well-defined paracrine factor that promotes SSC self-renewal [54]. GDNF signaling acts via the RET tyrosine kinase [55] and requires a ligand-specific co-receptor GFRα1 [56] in mouse SSCs [57]. Evidences have shown that through the PI3K/AKT-dependent pathway [58] or the SRC family kinase (SFK) pathway [59], GDNF regulates the expression of the transcription factors B cell CLL/lymphoma 6 member B (BCL6B), ETS variant 5 (ETV5), DNA-binding protein 4 (ID4), LIM homeobox 1 (LHX1) and POU class 3 homeobox 1 (POU3F1) to drive SSC self-renewal [59].

**Fig. 2:**
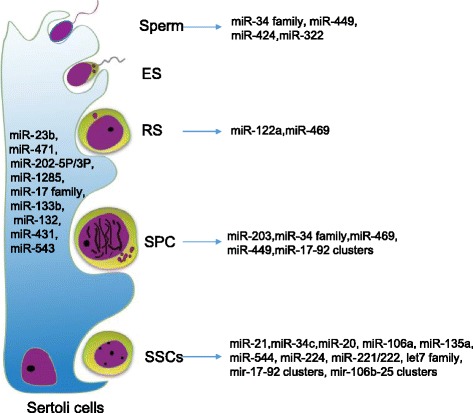
The expression of associate miRNAs in testicular cells

miRNAs conduce maintenance of the pool of SSCs. It has been shown that *miR-20* along with *miR-21, −34c, −135a, −146a, −182, −183, −204, −465a-3p, −465b-3p, −465c-3p, −465c-5p* and −*544* were preferentially expressed in the SSC-enriched population (Fig. 2) [60, 61]. Importantly, *miR-20, miR-21* and *miR-106a* contribute to maintenance of mouse SSC homeostasis [61]. *miR-135a* mediates the maintenance of rat SSCs by regulating FOXO1 that promotes high levels of Ret protein on the cell surface of SSCs [62]. Moreover, *miR-544* regulates self-renewal of goat SSCs by targeting the promyelocytic leukemia zinc finger gene (PLZF), which is the first transcription factor to be identified as being involved in SSC self-renewal [63]. Similarly, *miR-224* regulates mouse SSC self-renewal via modulating PLZF and GFRα1 [64]. Interestingly, *miR-34c* is expressed in goat SSCs and promotes SSC apoptosis in a p53-depemdent manner [65]. Recently, it was found that *miR-204* was involved in the regulation of dairy goat SSC proliferation via targeting Sirt1 [66]. Collectively, miRNAs are involved in regulating SSCs fate.

On the other hand, some miRNAs have been identified to mediate spermatogonia differentiation. It is well-known that retinoic acid (RA) directs the sequential programs of spermatogonial differentiation and the entry into meiosis [67, 68]. *miR-146* [69], *miR-let7* family miRNAs [70], *miR-17-92* and *miR-106b-25* clusters [71] are downregulated during RA-induced spermatogonial differentiation. Importantly, male germ cell-specific knockout of *miR-17-92* cluster resulted in the reduced number of SSCs and spermatogonia, and impaired spermatogenesis [71, 72]. Interestingly, exposure to RA downregulates *miR-221/222* expression, while GDNF upregulates *miR-221/222* abundance. Over-expression of *miR-221/222* in undifferentiated spermatogonia made them resisting to RA-induced transition into c-kit-positive differentiated spermatogonia [73]. In addition, *miR-34c* promotes SSC differentiation and meiosis process by targeting NANOS2 and up-regulating meiosis regulated genes *Stra8, Scp3* and *Dazl* [74]. Taken together, miRNAs are related to the post-transcriptional regulation of spermatogonia differentiation.

#### The roles of miRNAs in meiosis and spermiogenesis

Growing evidences have also demonstrated that specific miRNAs regulate meiosis (Fig. 2). The expression of *miR-449* cluster is abundant and is upregulated upon meiotic initiation during testis development and in adult testes. The expression pattern of the *miR-449* cluster is similar to that of *miR-34b/c*. Moreover, *miR-34b/c* and *miR-449* cluster share the same seed region and thus target same sets of mRNAs [75–78]. Depletion of either *miR-34* cluster or *miR-449* cluster displays no apparent defect in male germ cell development. However, simultaneous knockout of these two clusters led to sexually dimorphic and infertility, suggesting that *miR-34b/c* and the *miR-449* cluster function redundantly in the regulation of spermatogenesis [71]. Furthermore, *miR-18*, one of the *miR-17-92* cluster, is abundantly expressed in spermatocytes. *miR-18* targets heat shock factor2 (Hsf2), which is a critical transcription factor for spermatogenesis [79]. Finally, *miR-34b-5p* regulates meiotic progression by targeting *Cdk6* [80].

A unique chromatin remodelling occurs during spermatogenesis when histones are replaced by DNA packing proteins, such as transition proteins (TPs) and protamines (PRMs), which are exclusive to male germ cells [81, 82]. In the post-mitotic germ cells, the timely expression of TPs and PRMs is prerequisite for compaction and condensation of chromatin during spermiogenesis [83]. To secure this timed expression pattern, *Tp* and *Prm* are subjected to an efficiently post-transcriptional control. It has been demonstrated that *miR-469* suppresses the translation of TP2 and PRM2 by targeting mRNA of *Tp2* and *Prm*2 in pachytene spermatocytes and round spermatids [84]. On the contrary, *miR-122a* that is abundantly expressed in late-stage male germ cells reduces the *Tp2* mRNA expression by RNA cleavage [85].

Although the majority of miRNAs disappear during spermiogenesis, the sperm born miRNAs have also been demonstrated to play important roles. *miR-34* is present in mouse spermatozoa and zygotes but not in the oocytes or in embryos beyond the one-cell stage [86]. Upon fertilization, *miR-34c* is transferred from spermatozoa to zygote where it reduces the expression of Bcl-2 and p27, leading to S-phase entry and the first cleavage. Moreover, injection of *miR-34c* inhibitor into the zygotes inhibits DNA synthesis and suppresses the first cleavage division, suggesting that the sperm-borne *miR-34c* is required for zygote cleavage [86]. In addition, dysregulation of *miR-424/322* induces DNA double-strand breaks in spermatozoa [87]. Importantly, a set of sperm miRNAs are differentially expressed in asthenozoospermic and oligoastheno- zoospermic males compared with normozoospermic males [88, 89]. Furthermore, *miR-151a-5p* is abundant in severe asthenozoospermia cases compared with healthy controls and participates in mitochondrial biological functions [53, 90]. Therefore, specific miRNAs have been exploited as potential biomarkers for male factor infertility [91].

#### miRNAs in testicular somatic cells

Spermatogenesis is supported by the testicular Sertoli cells, peritubular myoid (PTM) cells and Leydig cells [92–94]. The extrinsic factors derived from these somatic cells trigger specific events in germ cells that dictate or influence spermatogenesis. It has been shown that miRNAs are highly abundant in Sertoli cells (Fig. 2) [12, 95, 96]. *MiR-133b* and *miR-202* are involved in pathogenesis of azoospermia or Sertoli-cell-only syndrome [97, 98]. Importantly,conditional depletion of *Dicer1* from Sertoli cells, using the Anti-Müllerian hormone (Amh) promoter-driven Cre in mice, results in disrupted spermatogenesis and progressive testis degeneration, indicating that miRNAs in Sertoli cells play critical roles in spermatogenesis [99, 100]. Specifically, *miR-133b* promotes the proliferation of human Sertoli cells by targeting GLI3 and mediating expression of Cyclin B1 and Cyclin D1 [97]. Moreover, *miR-762* promotes porcine immature Sertoli cell growth via the ring finger protein 4 (*RNF4*) [101].

FSH and androgens are fundamentally important for spermatogenesis. To elucidate the molecular mechanisms by which FSH and androgen act in the Sertoli cells, Nicholls et al. [102] investigated the expression and regulation of micro-RNAs (miRNAs). The authors have found that a subset of miRNAs were up-regulated after hormone suppression in rat model and in vitro culture of primary rat Sertoli cells. Interestingly, Pten, an intracellular phosphatase, and Eps15, a mediator of endocytosis, were down-regulated by the withdrawal of hormones [102]. In consistent with it, overexpression of *miR-23b* in vitro resulted in decreased translation of PTEN and EPS15 protein. Similarly, by using androgen suppression and androgen replacement, Chang et al. [53] identified that androgen regulated the expression of several microRNAs in mouse Sertoli cells [103]. One of the *miRNAs* targets found in this study is desmocollin-1 (*Dsc1*), which plays an essential role in cell-cell adhesion in epithelial cells [104]. On the other hand, elevated estradiol level is associated with male infertility [105]. Evidences indicate that estradiol regulates proliferation of Sertoli cells in a dose-dependent manner, in which *miR-17* family and *miR-1285* are involved in the regulation [106, 107]. Collectively, miRNA transcription is a new paradigm in the hormone dependence of spermatogenesis.

Leydig cells are responsible for androgen production that is essential for sperm production [108]. Basic fibroblast growth factor (bFGF) promotes the development of stem Leydig cells and inhibits LH-stimulated androgen production by regulating miRNAs [109]. Interestingly, *miR-140-5p/140-3p* control mouse Leydig cell numbers in the developing testis. Deletion of *miR-140-5p/miR-140-3p* results in an increase of number of Leydig cells, indicating that the miRNAs are likely to regulate the expression of factors produced by Sertoli cells that regulate differentiation of Leydig cells [110].

Collectively, these findings indicate that miRNAs regulate the development and functions of Sertoli cells and Leydig cells, which create the niche for SSCs and thus provide structural and nutritional support for germ cells. Therefore, miRNAs in somatic cells play important roles in spermatogenesis.

## Conclusion and perspectives

Extensive and accurate regulation of gene expression is prerequisite for spermatogenesis. miRNAs are expressed in a cell-specific or stage-specific manner during spermatogenesis. However, the roles and underlying mechanisms of many of those miRNAs in spermatogenesis remain largely unknown. Future studies should primarily focus on uncovering the roles of germ-cell specific miRNAs in spermatogenesis. The powerful single-cell small RNA sequencing would help to more accurately profile the miRNAs for certain type of germ cells. Meanwhile, the establishment of long-term culture of SSCs and in vitro induction of differentiation of male germ cells make it possible to elucidate the role of a certain miRNA or miRNA cluster in vitro. The application of CRISPR/Cas9 system and conditional knockout strategies would speed up the understanding of miRNA functions. Secondly, growing evidences have been demonstrated that some specific miRNAs are preferentially expressed in testicular somatic cells. But it is not clear whether these miRNAs act as secreted paracrine factors in the SSC niche, or whether they indirectly mediate the secretion of growth factors, GDNF for instance, which then affect germ cells. More somatic cell expressed miRNAs are needed to be functionally characterized. Thirdly, it has been demonstrated that some transcription factors promote SSC self-renewal (for example, BCL6B, BRACHYURY, ETV5, ID4, LHX1, and POU3F1), while several transcription factors stimulate spermatogonia differentiation (DMRT1, NGN3, SOHLH1, SOHLH2, SOX3, and STAT3) [111]. However, it is unclear which and how miRNA/miRNA cluster regulates the expression of these transcription factors. Fourthly, it has been discovered recently that RNA methylation is involved in pri-miRNA processing [28, 37], opening the door for exploring RNA methylation in the biogenesis and function of the miRNAs. Future research will pay increasing attention on the understanding of biological functions of epigenetic changes (or marks) during germ cell development. Finally, specific miRNAs in spermatozoa or seminal plasma will be exploited as potential biomarkers for male factor infertility. The annotation of the miRNAs and the elucidation of their regulating mechanisms in pathogenesis will provide insight into the etiology of male sterility and infertility. Together, uncovering these questions will shed new light on the pivotal roles of miRNA in spermatogenesis and fertility.

## Abbreviations

Aal: Aalign
Ad: Dark type A
AGO: Argonaute protein
ALKBH5: alkB homolog5
Apr: Apair
As: A single
ATF1: Activating transcription factor 1
bFGF: Basic fibroblast growth factor
DGCR8: DiGeorge syndrome critical region 8
DMRT1: Doublesex and mab-3 related transcription factor 1
Drosha: Drosha ribonuclease III
Dsc1: Desmocollin-1
E2F1: E2F transcription factor 1
ETV5: ETS variant 5
EXP5: Exportin5
FOXO1: Forkhead box protein O1
FSH: Follicle-stimulating hormone
FTO: Fat mass and obesity-associated protein (FTO)
GDNF: Glial cell line-derived factor
GFRα1: GDNF family receptor alpha-1
GLI3: GLI family zinc finger3
HNRNPA2B1: Heterogeneous nuclear ribonucleoprotein A2/B1
Hsf2: Heat shock factor2
LH: Luteinizing hormone
METTL3: Methyltransferase like 3
miRISC: miRNA-induced silencing complex
miRNA: Micro RNA
mTOR: Mechanistic target of rapamycin
PLZF: Promyelocytic leukemia zinc finger gene
Prm1: Protamine 1
RA: Retinoic acid
SR-BI: Scavenger receptor class B type I
SSCs: Spermatogonial Stem Cells
STAT3: Signal transducer and activator of transcription 3
Stra8: Retinoic acid 8
TPs: Transition proteins
